# Dr. Thomas M. Rochester

**Published:** 1903-08

**Authors:** 


Dr. Thomas M. Rochester, of Brooklyn, died of consumption, at Rochester, July 12, 1903, aged 45 years. Dr. Rochester graduated in medicine at the University of Buffalo in 1878, at which time he was a resident of Rochester, where his mother now resides, and at whose house he died. Soon after graduating he removed to Brooklyn, where he became a practitioner of prominence and took an active part in the medical societies of the city of his adoption. He was also a member of the Oxford, Marine and Field, and Reform Clubs. He was a great grandson of Colonel Nathan Rochester, the founder of the city to which he gave his name, and a cousin of Dr. DeLancey Rochester, of Buffalo. The deceased was a remarkably bright student and attracted attention at the time of his graduation, on account of the unusual proficiency of his attainments. He is lamented by a large number of attached relatives and friends.

